# Comparison of cardiac image-derived input functions for quantitative whole body [^18^F]FDG imaging with arterial blood sampling

**DOI:** 10.3389/fphys.2023.1074052

**Published:** 2023-03-22

**Authors:** Murray Bruce Reed, Godber Mathis Godbersen, Chrysoula Vraka, Ivo Rausch, Magdalena Ponce de León, Valentin Popper, Barbara Geist, Lukas Nics, Arkadiusz Komorowski, Georgios Karanikas, Thomas Beyer, Tatjana Traub-Weidinger, Andreas Hahn, Werner Langsteger, Marcus Hacker, Rupert Lanzenberger

**Affiliations:** ^1^ Department of Psychiatry and Psychotherapy, Medical University of Vienna, Vienna, Austria; ^2^ Department of Biomedical Imaging and Image-guided Therapy, Division of Nuclear Medicine, Medical University of Vienna, Vienna, Austria; ^3^ QIMP Team, Center for Medical Physics and Biomedical Engineering, Medical University of Vienna, Vienna, Austria

**Keywords:** arterial input function, positron emission tomography (PET), image-derived input function, kinetic modelling, [^18^F]2-fluoro-2-deoxy-D-glucose ([^18^F]FDG)

## Abstract

**Introduction:** Dynamic positron emission tomography (PET) and the application of kinetic models can provide important quantitative information based on its temporal information. This however requires arterial blood sampling, which can be challenging to acquire. Nowadays, state-of-the-art PET/CT systems offer fully automated, whole-body (WB) kinetic modelling protocols using image-derived input functions (IDIF) to replace arterial blood sampling. Here, we compared the validity of an automatic WB kinetic model protocol to the reference standard arterial input function (AIF) for both clinical and research settings.

**Methods:** Sixteen healthy participants underwent dynamic WB [^18^F]FDG scans using a continuous bed motion PET/CT system with simultaneous arterial blood sampling. Multiple processing pipelines that included automatic and manually generated IDIFs derived from the aorta and left ventricle, with and without motion correction were compared to the AIF. Subsequently generated quantitative images of glucose metabolism were compared to evaluate performance of the different input functions.

**Results:** We observed moderate to high correlations between IDIFs and the AIF regarding area under the curve (r = 0.49–0.89) as well as for the cerebral metabolic rate of glucose (CMRGlu) (r = 0.68–0.95). Manual placing of IDIFs and motion correction further improved their similarity to the AIF.

**Discussion:** In general, the automatic vendor protocol is a feasible approach for the quantification of CMRGlu for both, clinical and research settings where expertise or time is not available. However, we advise on a rigorous inspection of the placement of the volume of interest, the resulting IDIF, and the quantitative values to ensure valid interpretations. In protocols requiring longer scan times or where cohorts are prone to involuntary movement, manual IDIF definition with additional motion correction is recommended, as this has greater accuracy and reliability.

## 1 Introduction

Positron emission tomography (PET) imaging combined with [^18^F]2-fluoro-2-deoxy-D-glucose ([^18^F]FDG) is a widely used tool in research settings and clinical diagnostics (Fletcher et al., 2008; Gupta et al., 2011; Rischka et al., 2018; Hahn et al., 2020). The most common semi-quantitative metric that can be derived from static PET acquisitions is the standard uptake value (SUV), which is used as a surrogate of glucose metabolism for quantifying FDG uptake. Nevertheless, significant limitations of the accuracy of SUV remain. One of these limitations stems from the fact that SUV cannot provide a reliable measure of the tissue kinetics and does not account for the tracer levels in plasma (Huang, 2000; Boellaard, 2011).

Dynamic PET imaging provides spatio-temporal metabolic characteristics when combined with kinetic modelling methods and may exhibit greater robustness than simplistic SUV measures (Patlak et al., 1983; Wu et al., 2001; Cheebsumon et al., 2011). A full kinetic analysis comprises the solving of differential equations and on the voxel-level variance can be high due to increased noise which leads to less reliable parameter estimates and thus an inconsistent kinetic model. Therefore, graphical methods such as Patlak analyses are being widely used in both clinical and research settings due to its robustness and simplicity. This is particularly feasible for [^18^F]FDG as the radiotracer exhibits almost irreversible kinetics within a common scan time of less than 60 min. Furthermore, studies have shown that glucose metabolism estimated with the Patlak plot represents a more accurate and robust index of glucose metabolic rate when compared to SUV (Freedman et al., 2003).

Another disadvantage of modelling procedures is the requirement of invasive blood sampling to produce an accurate arterial plasma input function (AIF). Image derived input functions (IDIFs) represent a promising non-invasive alternative, aiming to extract the AIF from a suitably large region containing a robust blood pool in PET images and may be supplemented with venous blood to improve accuracy to the IDIF (Hahn et al., 2012, 2013). To date, whole body (WB) PET scans were acquired using a multibed, multipass protocol whereby each bed position requires scan times of 2–5 min (Boellaard et al., 2015). This however decreases temporal resolution greatly and thus affects the accuracy of kinetic modelling. The introduction of WB continuous bed motion (CBM) protocols utilizing state-of-the-art PET/CT systems offer a promising solution to these issues. Here, dynamic PET data can be acquired with increased spatio-temporal resolution while still keeping the kinetic information intact (Osborne et al., 2015; Osborne and Acuff, 2016). This is achieved by combining a short dynamic scan over the cardiac region to automatically acquire the IDIF and subsequent multiple WB sweeps (van Sluis et al., 2021). Combined with a high temporal resolution at the start of the protocol this further mitigates spillover effects. This then disseminates to improved estimation of the input function and outcome parameters (Viswanath et al., 2020) and thus better quantitative images (Wang et al., 2020; Zhang et al., 2020; Dimitrakopoulou-Strauss et al., 2021). Another option used in brain activation studies would be using a reference region to normalize the PET image; this however is only semi-quantitative, thus limiting accuracy (Clark et al., 1985; Dukart et al., 2010; Hua et al., 2015).

In theory, such an approach would represent a clinically feasible protocol, which provides quantitative estimates of glucose metabolism and high subject throughput.

In this work, we aim to validate the automatically generated IDIFs derived from the aorta and left ventricle using a Biograph Vision Edge (Siemens Healthineers, Germany) PET/CT system and included vendor-specific software with its recommended settings to the reference standard AIF (https://www.siemens-healthineers.com/molecular-imaging/options-and-upgrades/software-applications/flowmotion-multiparametricpet-suite). Furthermore, IDIFs were also extracted manually and images were corrected for motion. Finally, brain glucose metabolism was computed with the Patlak plot using the automatic vendor-based approach as well as manually generated IDIFs and AIFs.

Although the validity of the IDIF, using various blood pools and tracers, has already been assessed (Van der Weerdt et al., 2001; Hahn et al., 2012, 2013; Sari et al., 2021), the comparison of a vendor specific, fully automated IDIF software has yet to be evaluated. As PET/CT scanners become more advanced and image quality improves, automatic quantification software solutions will become more commonplace. Therefore, it is paramount to assess the strengths and limitations of this type of solution. If the vendor specific software were proven to accurately match the AIF, it would greatly improve accessibility to molecular imaging research and reduce labor needed in a clinical setting. This comparison study aims to provide arguments to what extent the fully automated IDIF software is suitable for absolute quantification in both clinical and research settings. We used the cerebral metabolic rate of glucose (CMRGlu) and WB net influx constant Ki to assess the effects each input function has on tissue quantification.

## 2 Materials and methods

### 2.1 Participants and study design

Seventeen healthy participants were included in this study (mean age = 25 ± 4 years, 6 female) and underwent a single PET/CT scan. All participants underwent a standard medical examination at the initial screening visit, which included blood tests, electrocardiography, neurological testing and the Structural Clinical Interview for DSM-IV performed by an experienced psychiatrist. Female participants also underwent a urine pregnancy test at the screening visit and before the PET/CT scan. Exclusion criteria included current and previous (12 months) somatic, neurological or psychiatric disorders, current and previous substance abuse or psychotropic medication, current pregnancy or breast feeding and previous study-related radiation exposure in the past 10 years. After a detailed explanation of the study protocol, all participants gave written informed consent and were financially reimbursed for their participation. The study was approved by the Ethics Committee (ethics number: 2054/2020) of the Medical University of Vienna. Procedures were carried out in accordance with the Declaration of Helsinki.

### 2.2 PET/CT data acquisition

Synthesis of the radiotracer [^18^F]FDG was carried out as described previously (Rischka et al., 2018). The radiotracer was injected *via* the cubital vein as a bolus (5.1 MBq/kg in 10 mL over 1 min) using a perfusion pump (Syramed µSP6000, Arcomed, Regensdorf, Switzerland) and was kept in a wolfram shield to minimize radiation exposure. PET and CT data was acquired using a Siemens Biograph Vision 600 Edge (Siemens Healthineers, Germany); (axial FOV: 26.1 cm, sensitivity: 16.4 kcps/MBq, TOF resolution: 210 ps (van Sluis et al., 2019)), using a modified Siemens FlowMotion Multiparametric PET protocol. In detail, PET acquisition was started simultaneously with the intravenous bolus of [^18^F]FDG (mean ± std activity: 304 ± 142 MBq).

During the first 5 min, a cardiac region single-bed list-mode acquisition was attained (frames: 24 × 5 s, 6 × 10 s, 4 × 30 s) to determine the IDIF from 3 volumes of interest (VOIs) [left ventricle (LV), thoracic aorta and liver as control region]. Afterwards, a CMB WB PET scan was started ranging from head to middle intestines with the following frames: 8 × 120 s and 7 × 300 s, with a bed speed of 5.43 mm/s and 2.17 mm/s respectively. Both the single-bed list-mode acquisition and the WB PET data were reconstructed using the scanners reconstruction software with default settings: 3D-TOF OP-OSEM with 4 iterations, 5 subsets and no filtering into a 220 × 220 image matrix with a voxel size of 3.3 × 3.3 × 4 mm.

Low-dose CT scans (tube voltage: 120 kVp, tube current 20 mA, CareDose4D, CarekV) were acquired after expiration and breath-holding commands. The CT images were reconstructed with a voxel size of 0.98 × 0.98 × 4 mm.

A T1-weighted image was also acquired using a Siemens Prisma 3T MR system equipped with a 64-channel head coil with the following parameters: TE/TR = 2.95/2,300 ms, TI = 900 ms, flip angle = 9°, GRAPPA 2, 240 × 256 mm field of view, 176 slices, 1.05 × 1.05 × 1.20 mm. Coefficients of variation were estimated for multiple VOIs to determine the homogeneity of the activity in both cardiac and whole-body sequences.

### 2.3 Processing pipelines

**FIGURE 1:**
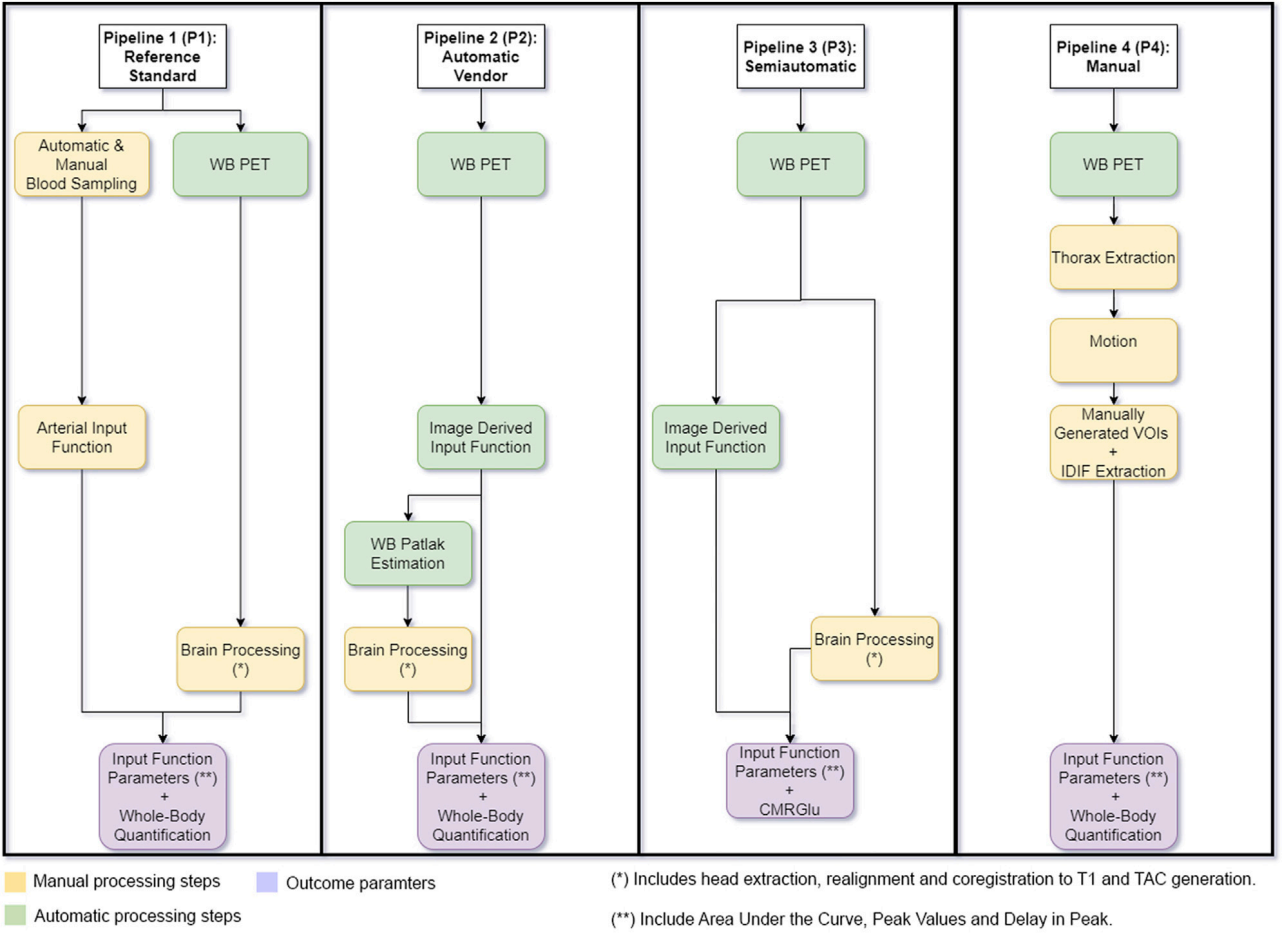
Graphical Overview of each processing pipeline used. Pipeline 1 represents the reference standard where arterial blood samples were used to generate cerebral metabolic rate of glucose (CMRGlu) values. Pipeline 2 represents the vendor implemented pipeline including image derived input function extraction and Patlak slope and intercept generation. CMRGlu was subsequently estimated manually. Pipeline 3 shows the semiautomatic processing pipeline where the automatic vendor derived input function was used to manually process CMRGlu estimates (the IDIF here is the same as in P2). Pipeline 4 includes manually placed volumes of interest used to extract the image derived input function. Whole-body images were then corrected for movement using a least squares approach and a 6 parameter rigid body spatial transformation as implemented in SPM12 and further calculation were done on both movement corrected data and compared to the arterial input function. Outcome estimates from each pipeline were compared to each other to assess similarity. Yellow boxes represent all manual processing steps, Green boxes represent all automatic vendor processing steps and purple signifies the pipeline outcome parameters.

Pipeline 1 (P1) represents the reference standard where the AIF was used to estimate CMRGlu values. Pipeline 2 (P2) represents the fully automatic vendor pipeline including image derived input function extraction from the left ventricle and descending thoracic aorta. WB Patlak slope and intercept generation were also performed by the vendor software. Pipeline 3 (P3) represents the semiautomatic processing pipeline where the automatic vendor derived input function was used for further manual Patlak modelling of CMRGlu. Pipeline 4 (P4) signifies a fully manual processing pipeline with a motion correction step applied before input function extraction. Here manual VOIs were placed as similar as possible to P2. Outcome estimates from each pipeline were compared to each other to assess differences between them, see Figure 1 for a graphical overview of each pipeline. There were no differences in preprocessing of the PET images. The quantification of the WB images for all participants was exemplarily restricted to the brain and subsequent brain regions to allow for a more accurate comparison between the pipelines.

### 2.4 Arterial blood sampling and AIF

During the first 5 min, blood was automatically sampled from the radial artery (4 mL/min Allogg, Mariefred, Sweden). Thereafter, manual samples were taken at 5, 10, 20, 40, 60 min. In these samples, plasma was separated from whole blood and activity of both was measured in a gamma counter (Wizward2, 3”, Perkin Elmer). To compute the AIF, the activities of the automatic and manual samples were combined and multiplied with the average plasma to whole-blood ratio, similar to (Hahn et al., 2020).

For the calculation of the area under the curve (AUC), the AIF was filtered to decrease noise levels present at the end of the automatic arterial blood sampling. In detail: from the start until the half-maximum value of the descending peak no data was filtered. Afterwards, a static average of 3 s was applied (Feng et al., 1993). Finally, the final samples corresponding to the manual ones were not changed.

For the quantification of CMRGlu, the AIF was modeled with the sum of three exponentials from the peak onwards in PMOD 4.2 (PMOD Technologies Ltd., Zurich, Switzerland; www.pmod.com).

### 2.5 Automatically generated Patlak protocol

As part of the Siemens FlowMotion Multiparametric PET protocol, three IDIF’s were automatically generated *via* Siemens deep learning software Automatic landmarking and parsing of human anatomy (ALPHA) by using the participants CT to define VOIs: left ventricle, descending thoracic aorta and liver as a control region, see Supplementary Figure S1 for the placement. These VOIs are then applied to the combined dynamic cardiac series to create input functions and subsequently generate the WB images of the net influx constant Ki (i.e., the slope resulting from the Patlak plot). Here by default, the scanner software uses the aorta VOI as the input function. Therefore, all quantified data was done using the aorta VOI and subsequently derived IDIF to reduce bias. The brain was extracted from the WB Ki image and spatial preprocessing was done in SPM12 build 7771 (The Wellcome Centre for Human Neuroimaging, www.fil.ion.ucl.ac.uk) using default parameters unless otherwise specified. Spatial preprocessing included co-registering the averaged extracted brain data to the T1-weighted image. Since the normalization procedure of SPM12 is optimized for MRI data the T1-weighted image was normalized to MNI-space and the resulting transformation matrices, (co-registration and normalization) were applied to the dynamic PET brain data (Gryglewski et al., 2017).

### 2.6 Manual extraction and movement correction

A volume of interest (VOI) (175 × 726 × 212 mm) was placed around the heart and surrounding structures and were extracted from each PET frame. These dynamic frames were then motion corrected using a least squares approach and a 6-parameter rigid body spatial transformation to the first acquired WB image in the series as implemented in SPM12. Thereafter, manually specified VOIs were positioned in the descending thoracic aorta and left ventricle with the participants CTs as a reference. Each VOI had the same dimensions and was placed in approximately the same position to the automatically generated VOIs for a more accurate comparison. In the case of the automatic VOIs being misplaced, the manual VOIs’ position was correctly placed in the middle of the blood pool to avoid any IDIF extraction biases. The VOIs were made as large as possible to avoid partial volume effects, spillover effects and interfering with the aortic/ventricle wall. The aorta VOI was created using a cylinder shape with a diameter of 3.3 mm and a length of 12 mm. The left ventricle VOI was extracted using a sphere VOI with a diameter of 9.9 mm. The mean activity in each VOI was extracted for each time point, representing the IDIFs.

### 2.7 Quantification of cerebral glucose metabolism

For comparison with the automatically generated metabolic images, all manual calculations of CMRGlu were also done using the aorta as input. Time activity curves (TACs) were extracted for 10 regions of the Harvard-Oxford atlas (frontal, temporal, parietal, occipital, cingulate, somatosensory, thalamus, striatum, amygdala/hippocampus and cerebellar gray matter). The Patlak plot as implemented in PMOD 4.2 was used for the quantification of Ki for the AIF, the manually derived IDIF and the automatically obtained IDIF CMRGlu was then calculated for all Ki outputs as follows:

$$CMRGlu=\frac{Ki^{*}{Plasma\;Glucose}_{i}}{Lumped\;Constant}$$

Where the lumped constant was set to 0.89 (Graham et al., 2002).

Whole-body glucose metabolic rates were calculated voxel-wise using the Patlak model with the AIF or IDIF as obtained from the vendor software (i.e., automatically extracted from the aorta) as well as a manually extracted IDIF from the left ventricle or aorta.

### 2.8 Statistical analysis

The similarity of both the automatically and manually generated IDIFs as compared to the gold standard of the AIF was assessed *via* regression analysis and spearman correlations. Here both the AUC and peak values were evaluated. Peak values and CMRGlu extracted from both IDIFs and AIF were compared to each other using the Wilcoxon signed rank test. Furthermore, the agreement between the automatically generated CMRGlu images was compared to the manually quantified ones using regression analyses. All statistical tests were performed using MATLAB R2018b and the significance level was set to *p* < 0.025 (two tailed). To correct for the number of regions in the Harvard-Oxford atlas, all tests were adjusted for multiple comparisons *via* the Bonferroni procedure. Mean absolute percentage errors of all voxels in the WB Ki images were estimated and compared between the manually and automatically extracted IDIFs to the AIF.

## 3 Results

**FIGURE 2:**
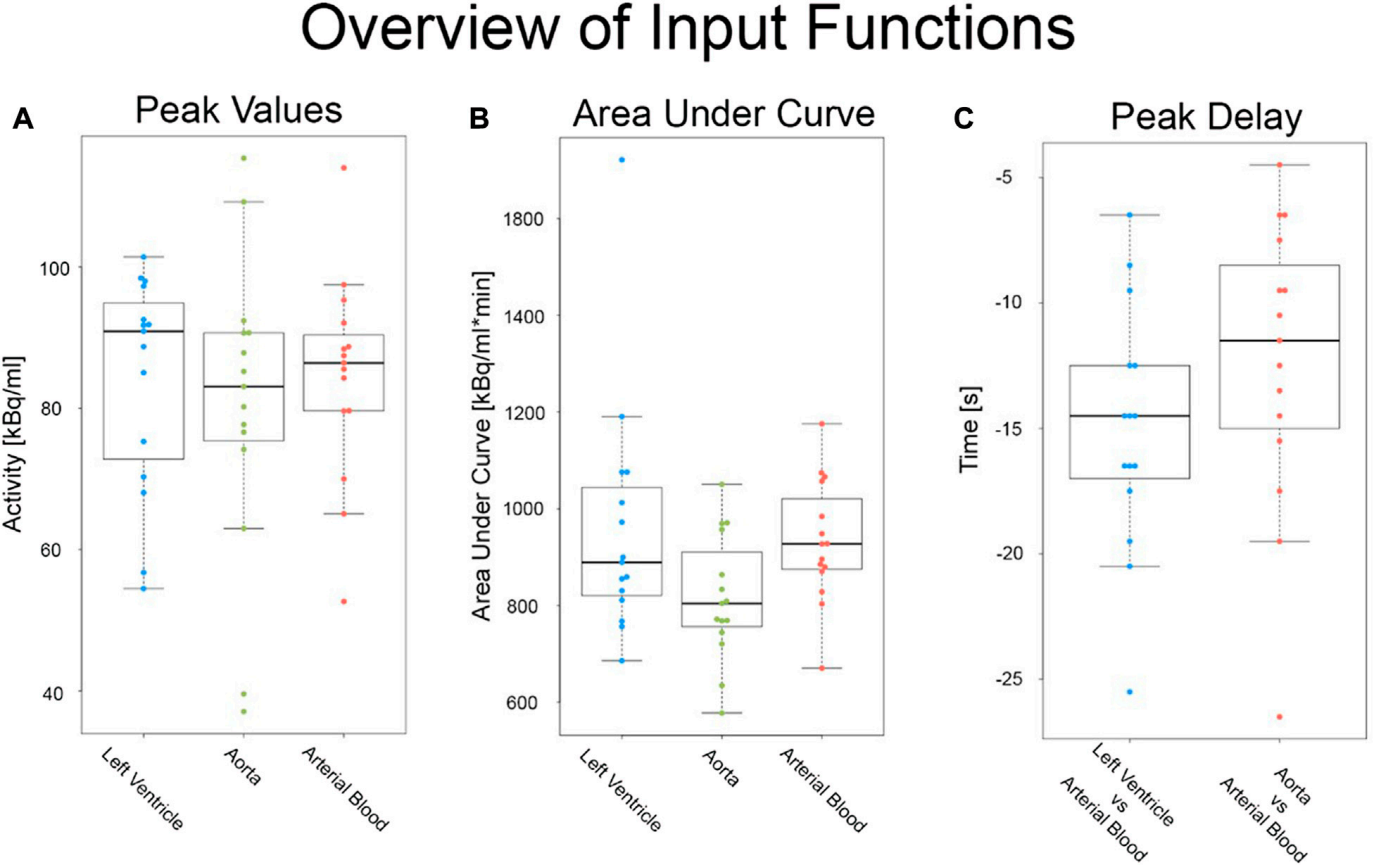
Overview of all input functions before motion correction: Comparison of raw **(A)** peak values, **(B)** area under the curve and **(C)** peak delay metrics in both the arterial and image derived input functions from the automatic vendor pipeline.

Out of the seventeen participants recruited, one was dropped due to a failure in arterial sampling. Furthermore, the vendor-based automatic generation of all three VOIs was successful for only 15 participants. Visual inspection of the automatic vendor generated VOIs showed a suboptimal placement of both the aorta and LV regions. The center of the aorta VOI was frequently located in the aortic wall, whereas the LV was seen over the ventricle wall. Furthermore, the peak value in the automatically generated aorta IDIF was on average lower than that of the AIF and manually placed VOIs. The LV had higher peak values than AIF, but not statistically significantly, (p _LV/blood_ = 0.45 and p _aorta/blood_ = 0.27, Figure 2A), while the AUC of both scanner generated IDIFs were lower than the AIF (Figure 2B). Finally, the LV IDIF peaked on average 15 s ± 5 s earlier than the AIF. Similarly, the aorta IDIF peaked on average 12 s ± 6 s earlier to the AIF, see Figure 2C. Supplementary Table S1 shows the coefficient of variance for organs of interest.

### 3.1 Comparison of input functions

**FIGURE 3:**
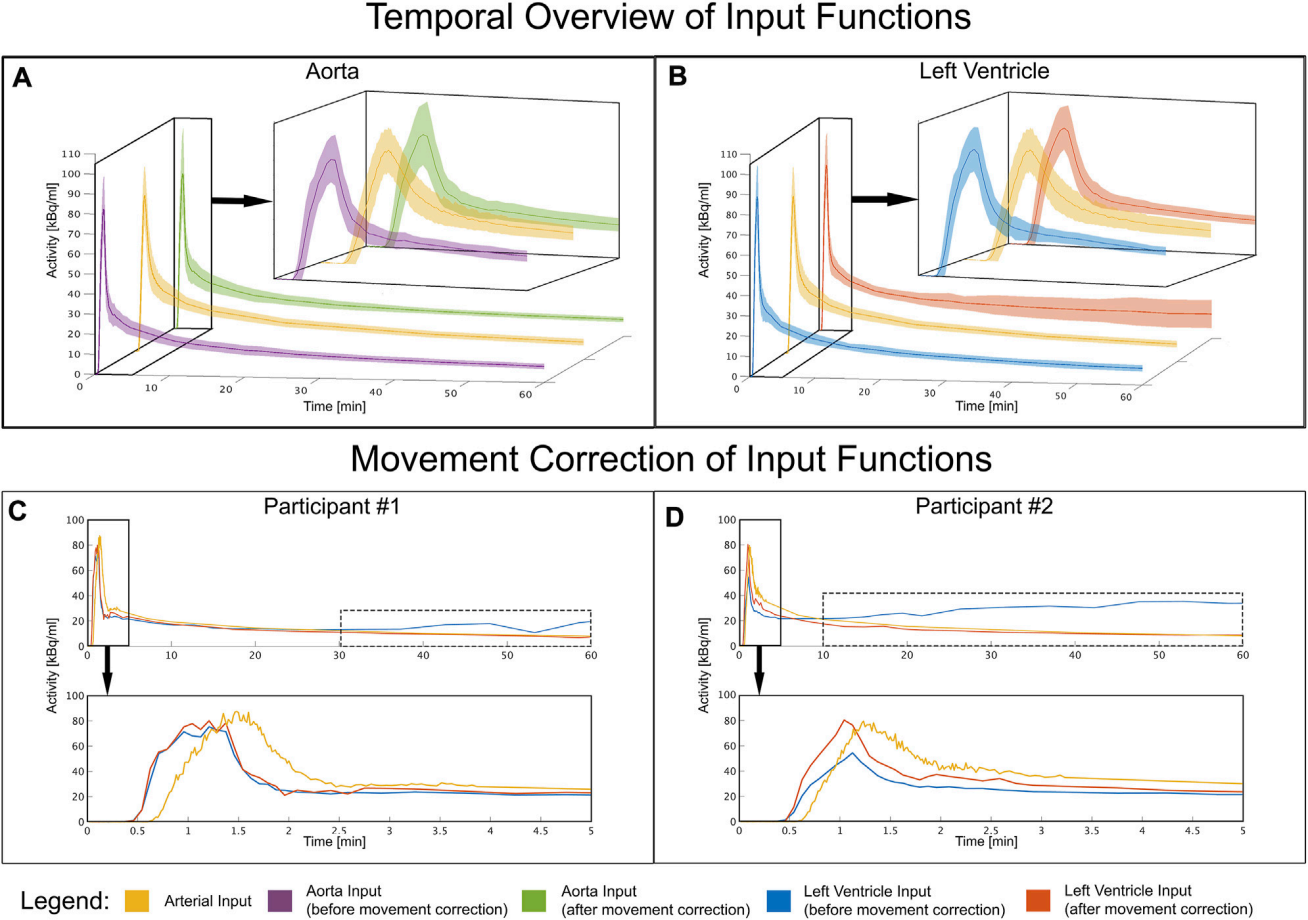
Temporal overview of each input function: (**A)** Mean and standard deviation time course of the image derived input function before and after movement correction in comparison to the arterial input function of all participants. The aorta shows a stable and concurrent input function compared to arterial blood both before and after movement correction. **(B)** Mean and standard deviation time course extracted from the left ventricle for all participants **(C–D)** Shows the input functions of two participants affected by movement problems. Towards the end of the measurement, the variance increases due to movement which is indicated by the dotted box. This was corrected by applying movement correction. Time course extracted from the left ventricle of two very movement intense participants and how motion correction counters these effects, to better match the arterial input function.

Figures 3A, B shows a visual comparison of the time course of all input functions of the aorta and LV for both the first 5min and the entire measurement. Figures 3C, D highlight the LV IDIF from P2 of two participants’ that were affected by both involuntary and voluntary motion. After motion correction (P4) was applied the LV IDIF more closely resembled that of the AIF.

**FIGURE 4:**
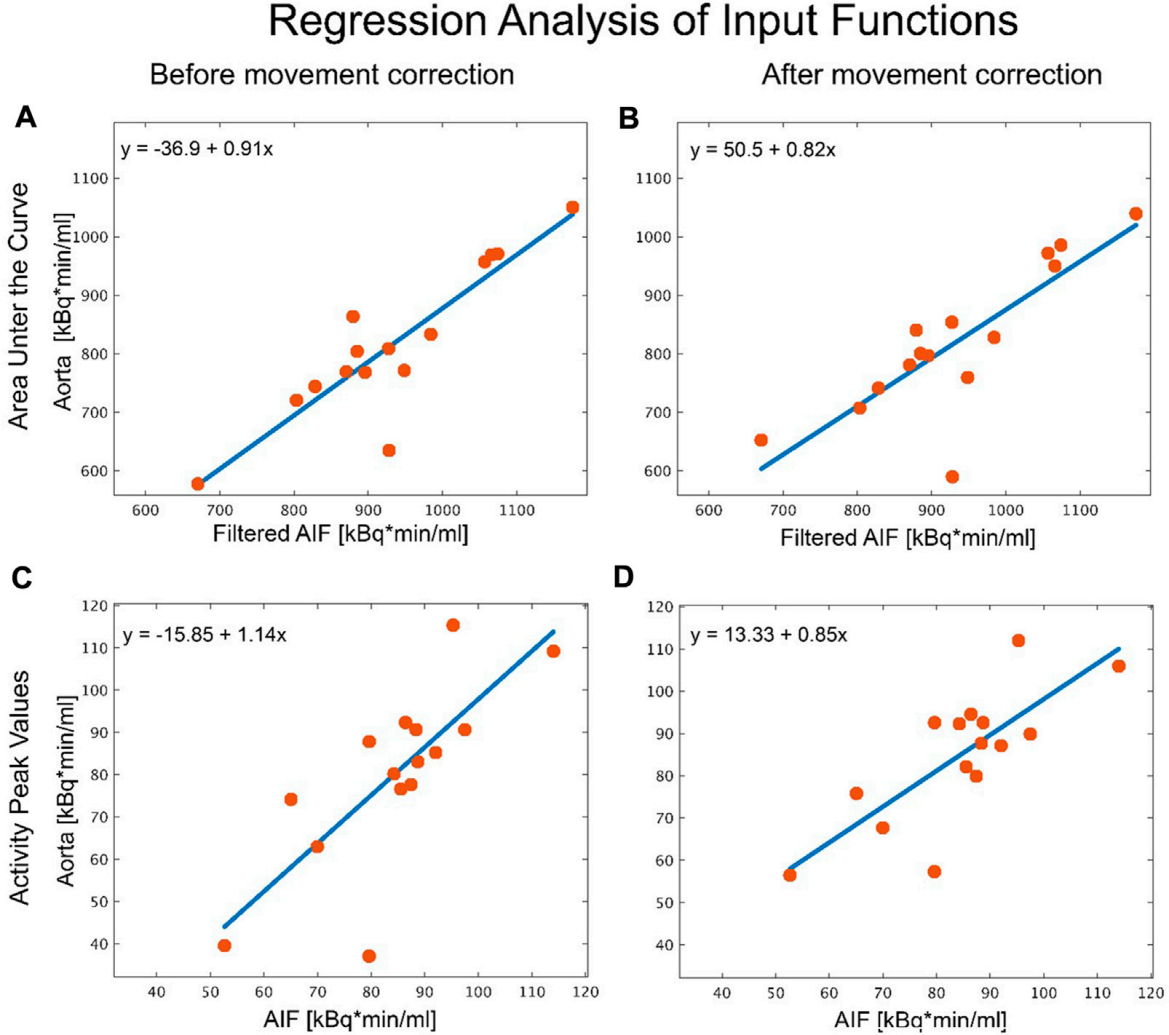
Analysis of the different aorta input functions. Regression analysis comparing peak values and area under the curve (AUC) of the arterial input function (gold standard) to an image derived input function (IDIF) placed in the aorta before and after movement correction. **(A)** AUC analysis of the automatic vendor generated IDIF in the aorta before movement correction vs. the AIF. **(B)** AUC analysis of the manually placed volume of interest (VOI) in the aorta after movement correction vs. the AIF. **(C)** As in **(A)**, regression analysis of peak values in the automatic vendor generated VOI vs. AIF before and **(D)** in the manually created VOI after correcting for movement vs. AIF. The source of the discrepancy between the IDIF and AIF method for the two outliers stem from misplaced VOIs when leading to discrepancies in the IDIF, where movement correction had no effect, due to it being incorrectly placed from the beginning.

AUC of the AIF displayed a moderate similarity with the LV IDIF from P2 (r = 0.49, *p* = 0.06) but strong agreement with the aorta IDIF from P2 (r = 0.89, *p* < 0.001, Figure 4A). After motion correction the similarity improved for the left LV IDIF from P3 (r = 0.97, *p* < 0.001) but had little effect on the aorta IDIF from P3 (Figure 4B). Similarly to the AUC, peak values between the AIF and aorta IDIF from P3 (r = 0.77, *p* < 0.001) and LV (r = 0.78, *p* < 0.001) showed a good agreement after movement correction (Figures 4C, D). Table 1 shows an overview of all comparisons between IDIF vs. AIF before (Table 1a), after (Table 1b) movement correction and a direct differentiation between IDIFs before and after movement correction (Table 1c).

**TABLE 1:** Overview of correlation and regression parameters between each input function. **(A) Shows comparisons using peak values and area under the curve of both image derived input functions to the arterial function before and (B) after movement correction.** **(C) Indicates a direct comparison between peak values and area under the curve before and after movement correction. The best correlation values before and after movement correction are depicted in bold. P3 was not included as the IDIF is the same as in P2.**

(a) Original vendor derived (P2)					
Comparison	Variable	Intercept	Slope	r	*p*-value
Aorta vs AIF	peak values	−15.8588	1.1374	0.7668	0.0009
Left ventricle vs AIF	peak values	14.1014	0.8284	0.7801	0.0006
Aorta vs AIF	AUC	−36.9029	0.9146	**0.8878**	<0.0001
Left ventricle vs AIF	AUC	47.9609	0.9779	0.4897	0.0639

(b) Manual VOI Placement including motion correction (P4)					
Comparison	Variable	Intercept	Slope	r	*p*-value
Aorta vs AIF	peak values	22.0767	0.8362	0.7900	0.0005
Left ventricle vs AIF	peak values	13.3300	0.8480	0.7775	0.0006
Aorta vs AIF	AUC	50.5049	0.8247	0.8228	0.0002
Left ventricle vs AIF	AUC	41.4682	0.8838	**0.9660**	<0.0001

(c) Movement correction (before vs. after)					
Comparison	Variable	Intercept	Slope	r	*p*-value
Left ventricle	peak values	17.8204	0.8908	0.8936	<0.0001
;Aorta	peak values	27.6595	0.7143	**0.9714**	<0.0001
Left ventricle	AUC	686.7625	0.1867	0.4075	0.1316
Aorta	AUC	46.5555	0.9473	**0.9737**	<0.0001

### 3.2 Quantification with different input functions

**FIGURE 5:**
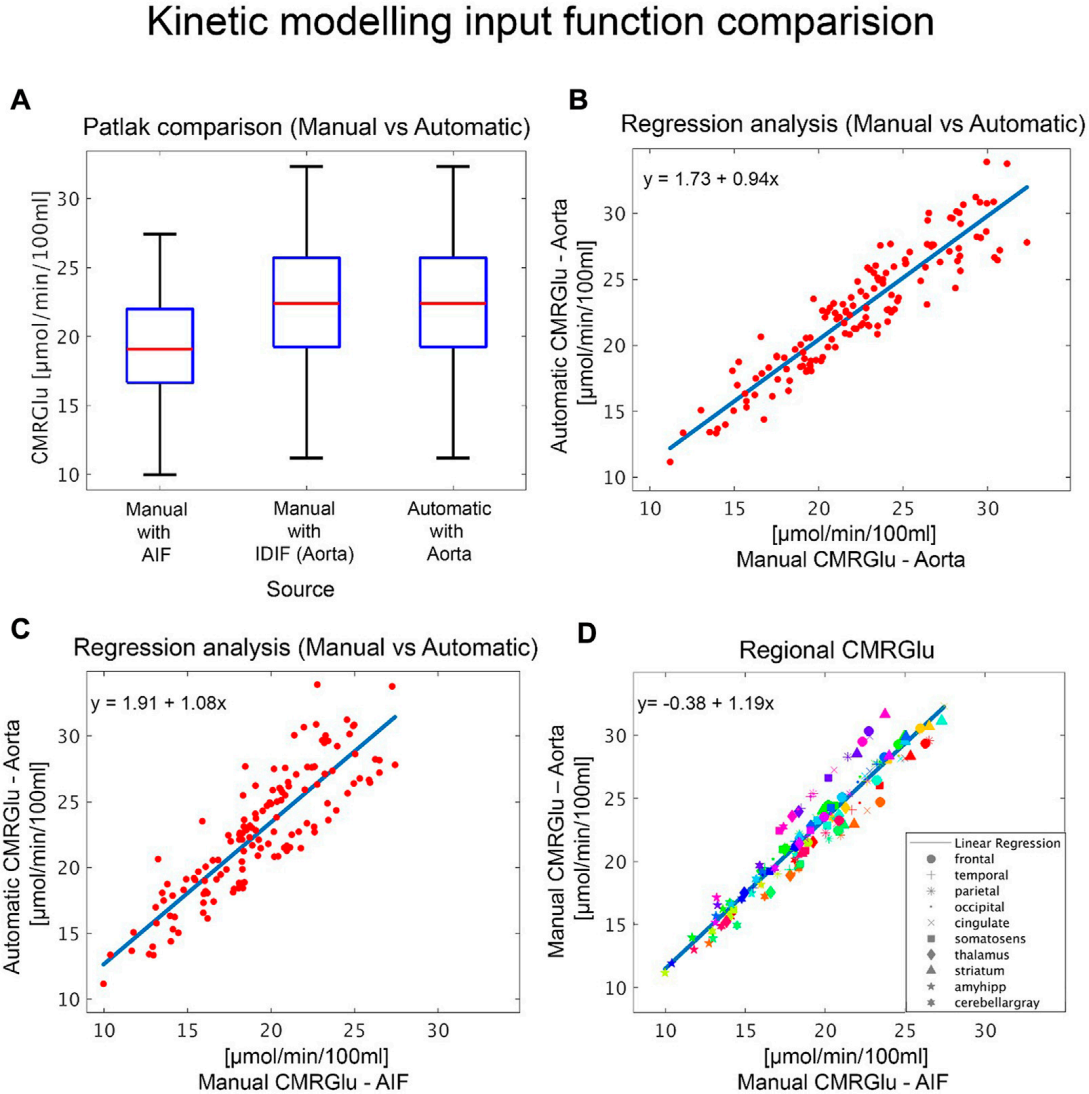
Comparison of kinetic modelling outputs: Quantification of glucose metabolism with different input functions. **(A)** Boxplots show overall cerebral metabolic rate of glucose (CMRGlu) values for the different input functions modelled either manually or automatically *via* the vendor software. **(B–C)** Regression analyses comparing CMRGlu obtained from the automatically generated aorta IDIF with **(B)** those from the manual aorta IDIF definition, modelling and motion correction (P4) and **(C)** the AIF. Subplot **(B–C)** plots include values across all regions and subjects. **(D)** Regional CMRGlu uptake comparison between the AIF and IDIF(Aorta). Here the different colors represent each participant, and each symbol represents the regions of interest, listed in the legend.

**FIGURE 6:**
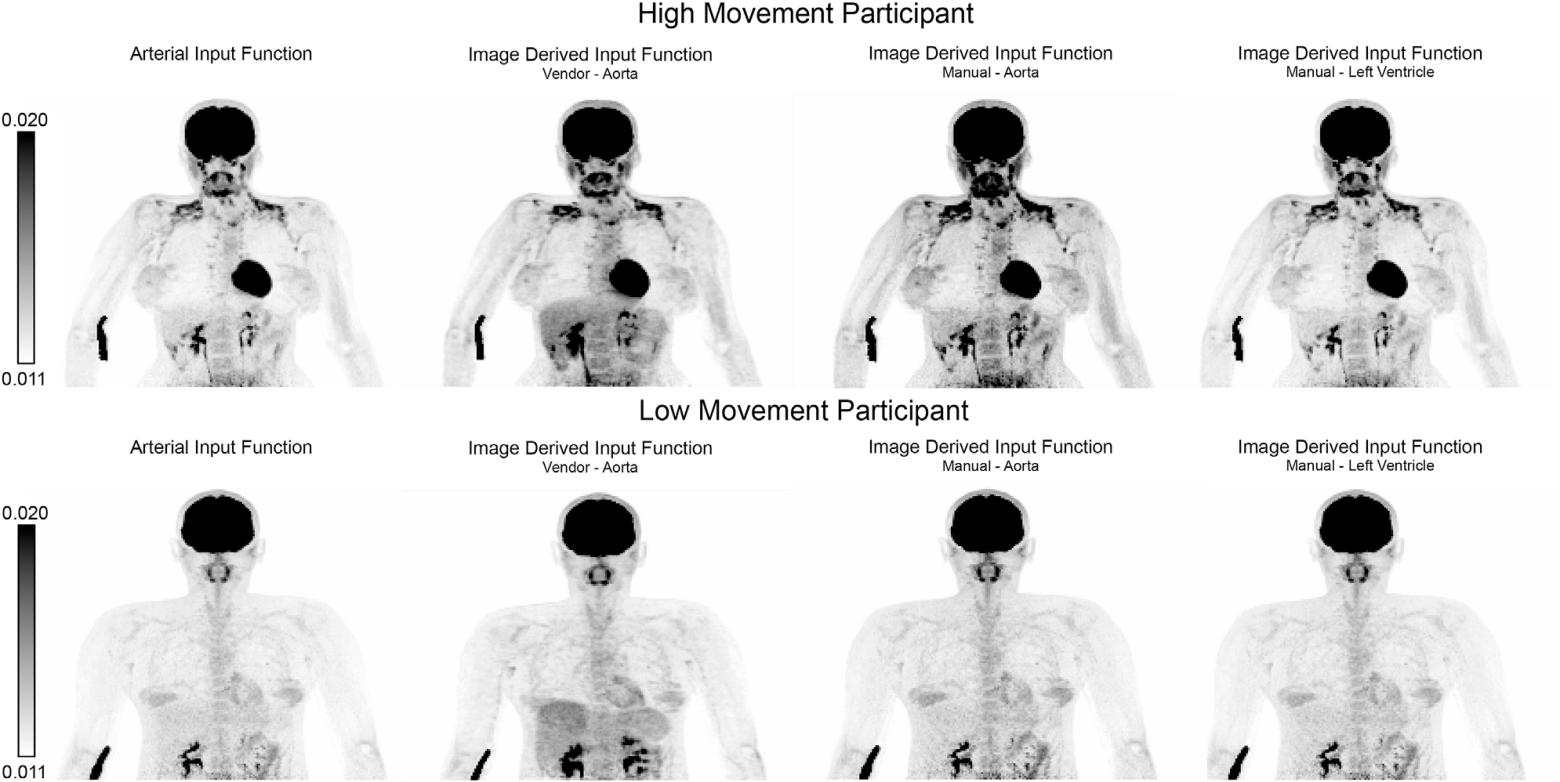
Qualitative comparison of whole-body quantitative outputs. Maximum intensity projections of whole-body quantified net influx constant (Ki) images derived from different input functions for a representative high and low movement participant. Quantified images were derived using (from left to right): arterial input function (gold standard, P1), automatic vendor image derived input function (IDIF) extracted from the aorta (P2), manually extracted aorta IDIF and left ventricle (P4).

The CMRGlu estimated for each brain region using the AIF (P1) was generally lower but not significantly different (p_Bonferroni_ > 0.1) than that of the manually estimated with PMOD (Figures 5A, C) (P3). Both IDIFs showed similar CMRGlu values (Figure 5B) (P2 and P3). When comparing CMRGlu values derived from the automatic vendor generated IDIF (P2) to the AIF (P1), gray matter CMRGlu values were greater in P1 than in P2. Specifically in the cingulate, frontal and temporal significant regional differences (p_Bonferroni_ < 0.001), see Figure 5D. An overview of correlation and regression analyses between the automatic LV and aorta IDIF from P2, aorta IDIF from P3 and AIF can be found in Table 2. Manually defined IDIFs from P4 had a lower error compared to the AIF for both left ventricle and aorta than the automatically extracted IDIF from the aorta (P2) in both high and low movement participants, see Table 3 and Figure 6 for detailed information.

**TABLE 2:** Overview of correlation and regression parameters from kinetic modelling. Comparison of regional cerebral metabolic rate (CMRGlu) uptake of all participants derived from automatic (vendor) to manual and arterial input functions. The best correlation values before and after movement correction are depicted in bold.

Automatic vs. semiautomatic input function derived quantitative values					
Comparison	Variable	Intercept	Slope	r	*p*-value
Manual IDIF(Aorta) vs AIF	CMRGlu	−0.3842	1.1906	**0.9551**	<0.0001
Manual IDIF(LV) vs AIF	CMRGlu	−0.1822	1.0036	0.6788	<0.0001
Vendor IDIF(Aorta) vs Manual IDIF(Aorta)	CMRGlu	1.7340	0.9362	**0.9278**	<0.0001
Vendor IDIF(Aorta) vs AIF	CMRGlu	1.9083	1.0771	0.8684	<0.0001

**TABLE 3:** Mean absolute percentage error of voxel-wise whole-body quantified [^18^F]FDG data for both automatic vendor image derived input function (IDIF) and the manually extracted IDIFs (P4) when compared to the gold standard: arterial input function for both a high and low movement participant.

Mean absolute percentage error [in %]		
	Low movement participant	High movement participant
Vendor Aorta	24.07	38.00
Manual Aorta	1.03	2.16
Manual Left Ventricle	0.66	2.20

## 4 Discussion

The purpose of this study was to assess the validity of a fully automatic IDIF and the resulting quantitative values of CMRGlu and WB Ki using a WB CBM [^18^F]FDG PET/CT protocol to the reference standard of AIF. We observed that the vendor generated VOIs were not always optimally placed, resulting in suboptimal input function extraction. The left ventricle VOI was more susceptible to motion artifacts stemming not only from its cardiac function but also body movement. However, once corrected from above mentioned artifacts, it showed the best agreement to the AIF (Figure 3). Nevertheless, both quantitative values derived from IDIFs and the IDIFs themselves indicated a good match to the AIF. Furthermore, a simple solution to correct for motion which was applied after the scan resolves uncertainties in IDIF generation, which may represent an important benefit for long scan protocols and patient groups more prone to movement (Figures 3, 4; Table 2).

Firstly, the similarity between the automatically vendor generated IDIFs, namely, (aorta and left ventricle) were compared to the AIF. Overall, we observed a good agreement in both AUC and the peak values from all three sources. Both IDIFs displayed an earlier peak onset when compared to the AIF, which most probably emerges from anatomical distance between the heart and the radial artery. Furthermore, we observed that the IDIFs derived from the LV had a higher variance than those derived from the aorta. This can be attributed to the motion intense physiological left ventricular function of the heart as well as potential spillover effects from the adjacent myocardium. Peak values derived from the scanner-generated aorta IDIF were generally lower than both the LV and AIF but more stable than the LV. The differences between the automatic pipeline when compared to the other 3 could be in part attributed to the automatic misplacement of both the aorta and LV VOIs. However, this was not the case for the manually derived aorta IDIF. Previous studies suggest that the aorta is a more robust option for the IDIF (Sari et al., 2021), which is also supported by our observations (Table 1c) as it is less susceptible to movement artifacts. Visual inspection of the automatic vendor generated VOI placement revealed sub-optimal placement for both the aorta and LV, which influences not only IDIF extraction but also the quantitative values generated. Similar peak and onset values extracted from the aorta and LV IDIFs were also found when using the new high-performance uEXPLORER scanner (Zhang et al., 2020). Zhang et al. also show that cardiac motion in the LV affects the TAC extraction, in their analysis the aorta was selected as the TAC extraction was not as affected by motion. They further compare smaller VOIs like the carotid, brachial and femoral arteries and show that these VOIs are affected by dispersion and partial-volume effects due to their size (Zhang et al., 2020).

Subject motion during scans can severely affect the placement of regions used for IDIF extraction, which in turn will affect modelling outcomes. For graphical analysis using the Patlak plot an incorrectly defined IDIF will affect the area under the curve and thus directly bias CMRGlu values (Yao et al., 2021). This can be seen in our results where we compared the CMRGlu values derived from both the manually and automatically defined IDIFs. Therefore, it is imperative to either choose a VOI less prone to motion artifacts or correct for such motion. One option to overcome this is to use a population derived input function, which by definition is not affected by motion (Rahmim et al., 2019; Naganawa et al., 2020; van Sluis et al., 2021). This method however is also not optimal as the input function is only scaled by the participants’ blood which must be extracted venously or arterially during the scan (Brock et al., 2005; Vriens et al., 2009). This can lead to bias (Zanotti-Fregonara et al., 2012; Naganawa et al., 2020) and may not account for individual as well as pathological variations in the shape of the input function. The low correlations between the LV and AIF AUC before movement correction are indicative of either misplaced VOIs or movement during the scan. This can also be seen when analyzing the IDIFs of individual subjects. We found manually placing the VOIs for each individual considerably improved the robustness of the IDIFs, as we were able to adjust for each individuals varying anatomy. By further correcting for movement artifacts, the LV IDIF displayed a better match to the AIF than the aorta. However, it is acknowledged that this may not be feasible in clinical settings. Similarly, using rigid-body transformations to correct for motion in the areas where the IDIF was extracted, the agreement between the IDIF and AIF further improved. Thus, the combination of manually placed VOIs and motion correction yielded the best results.

Since the peak values and AUC of the aorta IDIF were on average lower than that of the AIF, the CMRGlu was higher for both the scanner-generated and manually drawn aorta IDIF when compared to the AIF. Nevertheless, a high correlation between all three CMRGlu estimates was found (De Geus-Oei et al., 2006; Naganawa et al., 2020) and especially CMRGlu estimates for the grey matter were in line with previous literature (Liu et al., 2021; Sari et al., 2021). Of note, there were only a few exceptions to this, showing unphysiologically high Ki values. In most cases this can be corrected by reprocessing the data, which would require a trained technician to check each step of processing. This is time consuming and not feasible in many clinical scenarios. These problems did not appear when using manually drawn IDIFs or the AIF.

While dynamic PET imaging yields not only a more accurate clinical picture but also more spatio-temporal metabolic characteristics i.e., metabolic rate and distribution volume. This however comes with a cost, comprising prolonged scan time, complex protocols, longer image reconstruction times and invasive arterial blood sampling, which might not be tolerable for certain patient populations e.g., dementia patients. Therefore, static PET imaging utilizing SUV as a surrogate for metabolic rates has been used to reduce the workflow requirements of complex dynamic PET imaging. SUV is affected by several factors, including patient habitus, blood glucose levels and uptake time. All of these factors greatly reduce the information available for diagnosis (Braune et al., 2019).

We would like to acknowledge certain limitations of our work. Even though the AIF can be used as an input to the vendor pipeline, this data must be acquired from an external third party device. Thus, the comparison the AIF as an input for the automatically generated Ki images was not analyzed, consequently limiting the interpretability of the intrinsic, vendor specific quantification algorithm. Furthermore, as the vendor software comes as is, it was only possible to partly recreate a similar extraction and quantification pipeline, thus also limiting comparability between not only the IDIF’s but also the Ki image generation. In these instances, the vendor software was substituted with PMOD’s kinetic modelling software. Future development and validation of abovementioned approaches would greatly assist in WB group comparisons.

To summarize, for clinical use where the aim is to keep additional effort to a minimum, the automatic scanner protocol has many advantages. However, the shortcomings of such an automated method need to be taken into account before choosing the type of input function used and should clearly be stated to provide readers with a clear picture of possible limitations. We herein validate the use of absolutely quantified values of glucose metabolism directly obtained from a state-of-the-art PET system with the arterial input function reference standard. When using the automatic vendor software, an inspection of VOI placement, IDIF and quantitative image estimates should be done before further use. Finally, when using protocols that require longer scan times or patient cohorts prone to involuntary movement, manual VOI definition, additional movement correction and manual modelling for results yield more reliable and robust results.

## Data Availability

Due to data protection laws processed data is available from the authors upon reasonable request. Please contact rupert.lanzenberger@meduniwien.ac.at with any questions or requests.
