# Supplementary material for: Comparison of cardiac image-derived input functions for quantitative whole body [18F]FDG imaging with arterial blood sampling
Source: Front Physiol. 2023 Mar 22;14:1074052. doi: 10.3389/fphys.2023.1074052 (PMC10073457; doi:10.3389/fphys.2023.1074052)
Supplement: Supplementary file 1 [file DataSheet1.docx]

Supplementary Material


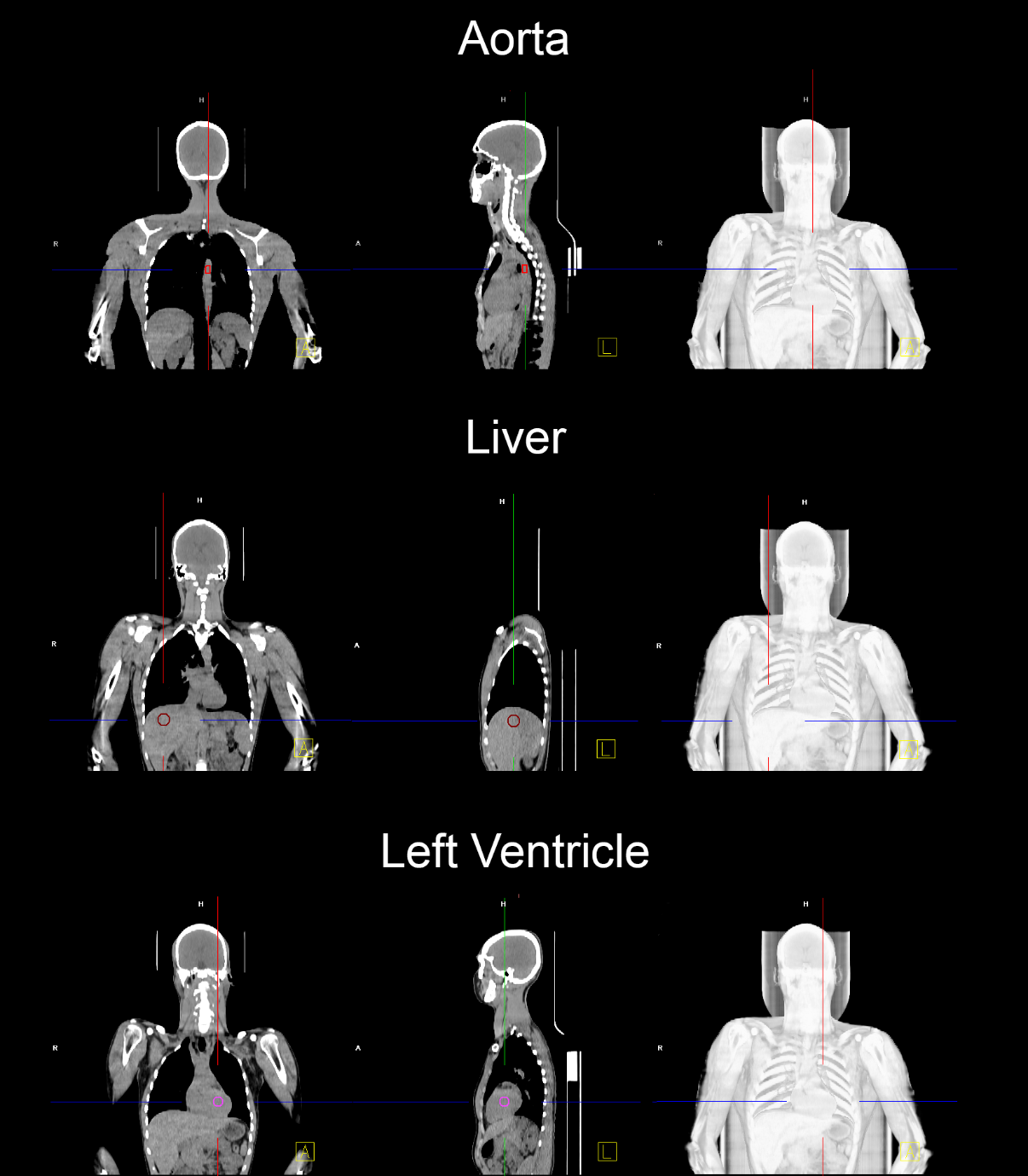


**Supplementary Figure S1:** Location of each automatically placed volume of interest by the vendor software.


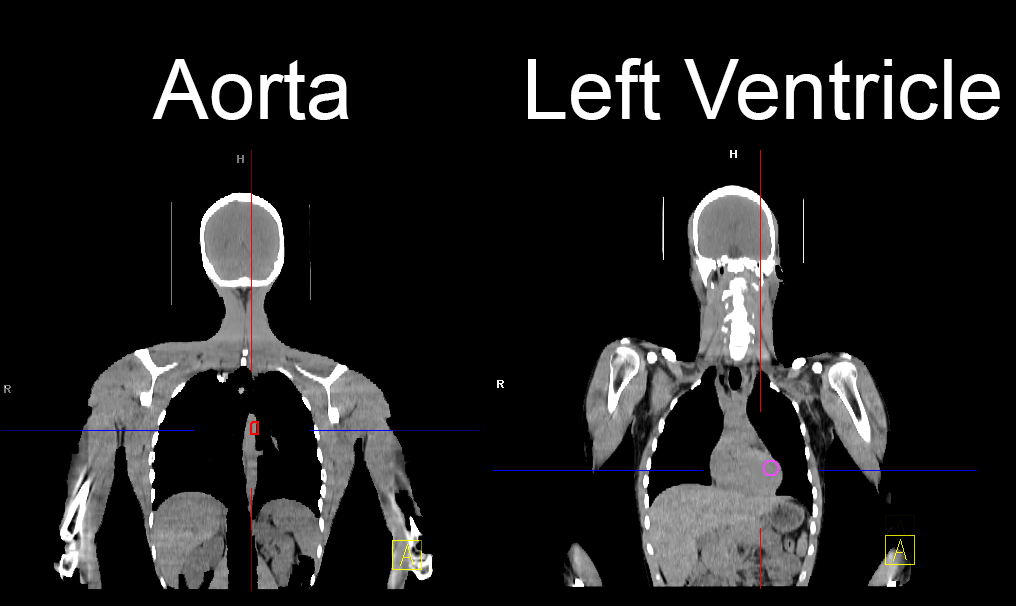


**Supplementary Figure S2**: Exemplary depiction of a misplaced VOI by the vendor-specific software.

| **Coefficient of Variation [kBq/cm^3^]** | | | | |
| --- | --- | --- | --- | --- |
| **VOI** | **Whole-body** | | **Cardiac** | |
|  | **Mean** | **SD** | **Mean** | **SD** |
| **Lung** | 0.49 | 0.19 | 0.47 | 0.08 |
| **Aorta** | 0.23 | 0.07 | 0.33 | 0.06 |
| **Left Ventricle** | 0.35 | 0.11 | 0.20 | 0.04 |
| **Brain** | 0.39 | 0.11 | - | - |
| **Liver** | 0.22 | 0.09 | 0.32 | 0.07 |

Table S1: Coefficients of variance extracted from organs of interest for both cardiac and whole-body sequences given in kBq/cm^3^ over all participants.
